# Excellent Carbonation Behavior of Rankinite Prepared by Calcining the C-S-H: Potential Recycling of Waste Concrete Powders for Prefabricated Building Products

**DOI:** 10.3390/ma11081474

**Published:** 2018-08-19

**Authors:** Kai Wang, Liang Ren, Luqing Yang

**Affiliations:** School of Civil and Architecture Engineering, East China Jiaotong University, Nanchang, 330013, China; renliang@163.com (L.R.); 2017018085213020@ecjtu.jx.cn (L.Y.)

**Keywords:** rankinite, carbonation, waste concrete, CO_2_

## Abstract

Pure rankinite (C_3_S_2_) was prepared by calcining a C-S-H gel precursor at a temperature of 1300 °C. The carbonation hardening behavior of the resulting rankinite was revealed by X-ray diffraction (XRD), Fourier transform-infrared (FT-IR) spectroscopy, thermogravimetry and differential thermal analysis (TG/DTA), and scanning electron microscope (SEM) coupled with energy dispersive spectrum (EDS). The results indicate that the pure rankinite can be easily prepared at a lower temperature. The cubic compressive strengths of the resulting rankinite samples reach a value of 62.5 MPa after 24 h of carbonation curing. The main carbonation products formed during the carbonation process are crystalline calcite, vaterite and highly polymerized amorphous silica gels. The formed carbonation products fill the pores and bind to each other, creating a dense microstructure, which contributes to the excellent mechanical strength. These results provide a novel insight into potential recycling of waste concrete powders for prefabricated building products with lower CO_2_ emissions.

## 1. Introduction

At present, China is at the peak of infrastructure construction. The number of new concrete buildings being constructed and old buildings being demolished is enormous. It has been conservatively estimated that China produces nearly 100 million tons of waste concrete each year [1]. Disposal of waste concrete not only requires a large amount of land resources, but also poses serious environmental issues. In this age of greater environmental awareness, an increased number of environmental laws and the desire reduce construction costs, the recycling of waste concrete into recycled aggregate concrete has many benefits, thus, making it an attractive option [2,3,4,5,6,7]. In this way, waste concrete is crushed and the aggregate is separated and recovered. However, the crushing process also produces 25–40% waste concrete powder (WCP). These powders are mainly C-S-H gels, with large specific surface areas, high water demands and have not been well reused in the past [8,9,10,11].

On the other hand, the use of some low-lime calcium silicate phases such as dicalcium silicate (C_2_S), rankinite (C_3_S_2_) and wollastonite (CS) to produce prefabricated buildings by carbonation, with significantly lower carbon dioxide emissions, is creating concern worldwide [12,13,14,15,16]. Rankinite (C_3_S_2_) is a low-lime calcium silicate phase. However, the traditional preparation method requires a higher calcination temperature (1460 °C) and cannot be synthesized easily [17].

In this paper, pure C_3_S_2_ minerals were prepared by calcining the prepared C-S-H gel precursor. The carbonation hardening behavior of the prepared C_3_S_2_ was revealed by X-ray diffraction (XRD), Fourier transform-infrared (FT-IR) spectroscopy, thermogravimetry and differential thermal analysis (TG/DTA), and scanning electron microscope (SEM) coupled with energy dispersive spectrum (EDS). The results provide a novel insight into the potential recycling of waste concrete powders for prefabricated building products, with lower CO_2_ emissions.

## 2. Materials and Methods

### 2.1. Preparation of C_3_S_2_

The C-S-H gel precursor was firstly prepared by exposing the mixtures of CaO and amorphous SiO_2_ (at 3:2 molar ratios) to a hydrothermal process. The water to solid ratio was 10 and the mixtures were sealed at 60 °C for 6 h to allow the complete reaction at ambient pressure. Then, the prepared C-S-H gel precursor was dried in a vacuum oven at 100 °C for 24 h. The dried C-S-H gel precursor was later calcined at 1300 °C for 2 h. Subsequently, the prepared C_3_S_2_ was cooled down to room temperature at a rapid cooling rate (approximately 500 °C/min) and ground for 20 min to achieve a Blaine fineness of 3970 cm^2^/g.

### 2.2. Carbonation of C_3_S_2_

C_3_S_2_ is a non-hydraulic mineral that does not set and harden when mixed with water. Thus, the resulting C_3_S_2_ powder was mixed with water at a water to solid ratio of 0.1 which is conducive to the carbonation reaction. Then, the wet mixtures were cast into a stainless steel mold (20 mm × 20 mm × 20 mm) and compacted at 5 MPa for 1 min. Thereafter, the compacted C_3_S_2_ samples were placed in a sealed stainless chamber at a temperature of 25 ± 2 °C, relative humidity of 70%, CO_2_ concentration of 99.9% and CO_2_ pressure of 0.3 MPa for 2, 5, 8 and 24 h, respectively.

### 2.3. Test Methods

#### 2.3.1. The Cubic Compressive Strength

The cubic compressive strength was measured using a universal testing machine with a deformation speed of 0.5 mm/min. Six cubic samples with a dimension of 20 mm × 20 mm × 20 mm were tested.

#### 2.3.2. X-ray Diffraction Analysis

The phase structure of the C_3_S_2_ phase before and after carbonation were characterized by powder X-ray diffraction on a Rigaku SmartLab X-ray diffractometer (Rigaku Corporation, Tokyo Akishima, Japan) with Cu K_α_ radiation (λ = 1.5406 Å). The X-ray tube was operated at 40 kV and 15 mA. The XRD patterns were recorded in the range of 10–55°.

#### 2.3.3. Fourier Transform-Infrared Spectroscopy

The FT-IR spectroscopy data of the C_3_S_2_ phase before and after carbonation was collected using a Bruker V70 Fourier transform infrared spectrometer (Bruker Corporation, Karlsruhe, Germany) with the KBr pellet technique, and the ranges of spectrograms were 1800–800 cm^−1^ at a resolution of 4 cm^−1^. Each spectrum presented in this paper is an average of six scans.

#### 2.3.4. Thermogravimetry and Differential Thermal Analysis

The TG/DTA tests were performed using a simultaneous thermal analyzer (BJ-HCT-3, Nanjing sangli electronic equipment factory, Nanjing, China). The sample weighing 20 mg was placed into a ceramic crucible, and then heated with a rate of 10 °C/min from 20 °C to 950 °C using an alumina reference material. N_2_ was used as purge gas during the TG/DTA tests.

#### 2.3.5. Scanning Electron Microscope

A small cut portion of the compacted C_3_S_2_ sample before and after carbonation was dried and epoxy impregnated, respectively. After impregnation, one of the surfaces was polished to a 0.5 micrometer finish. The polished surface was sputter coated with a thin layer of gold (Au) and examined under a SEM in backscattered mode. A Merlin Compact ultra-high-resolution field emission scanning electron microscope (FEI Corporation, Hillsboro, OR, USA) coupled with Oxford energy dispersive spectrum at 20 kV was used to acquire the images.

## 3. Results

### 3.1. The Cubic Compressive Strength

**Figure 1:**
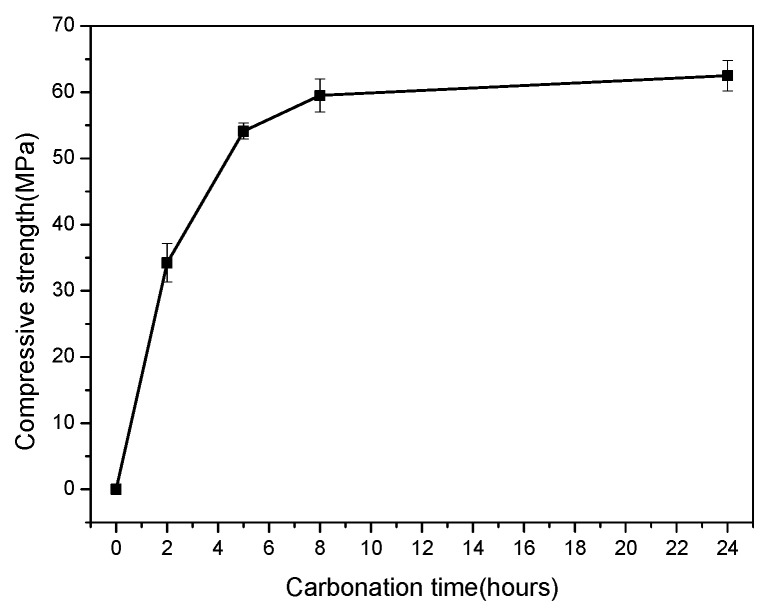
The compressive strength of compacted C_3_S_2_ samples with different carbonation times.

The cubic compressive strengths of the compacted C_3_S_2_ samples carbonated for 0, 2, 5, 8 and 24 h, respectively, are provided in Figure 1. The results show that compacted C_3_S_2_ samples can be rapidly hardened under carbonation conditions and reach a compressive strength of 62.5 MPa within 24 h. In addition, the strength development was mainly focused in the initial eight hours. These results indicate that C_3_S_2_ prepared from C-S-H gel precursors can achieve excellent strength after rapid carbonation curing, providing a novel insight into potential recycling of waste concrete powders for prefabricated building products with lower CO_2_ emissions.

### 3.2. Carbonation Products

#### 3.2.1. XRD Analysis

**Figure 2:**
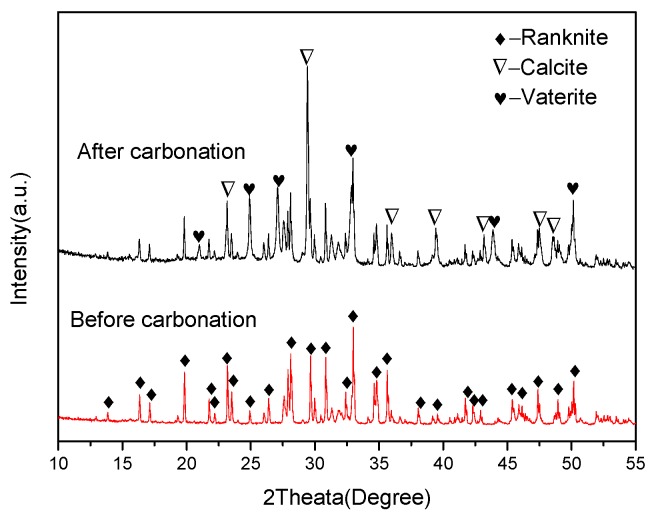
X-Ray Diffraction (XRD) patterns of the C_3_S_2_ phase before carbonation and carbonated for 24 h.

Figure 2 illustrates the XRD patterns of the C_3_S_2_ phase before carbonation and carbonated for 24 h. For the C_3_S_2_ phase before carbonation, the pattern matches well with the published XRD pattern for C_3_S_2_ [17,18]. It is indicated that the pure C_3_S_2_ phase can be easily prepared by calcining the C-S-H gel precursor at 1300 °C for two hours. After carbonation, the main crystalline carbonation products are calcite and vaterite and there are no diffraction peaks of silica, revealing that the silica formed during the carbonation is amorphous (SiO_2_ gels). These results are distinct from the results achieved by Qian [17] who believes that crystalline quartz and cristobalite are the main silica products formed after 24 h of carbonation. Moreover, some unreacted C_3_S_2_ phase still exists. The mass fractions of calcite, vaterite and unreacted C_3_S_2_ measured from the XRD pattern of the C_3_S_2_ phase after carbonation for 24 h by the Rietveld method are 44.7, 20.1 and 35.2%, respectively.

#### 3.2.2. FT-IR Analysis

**Figure 3:**
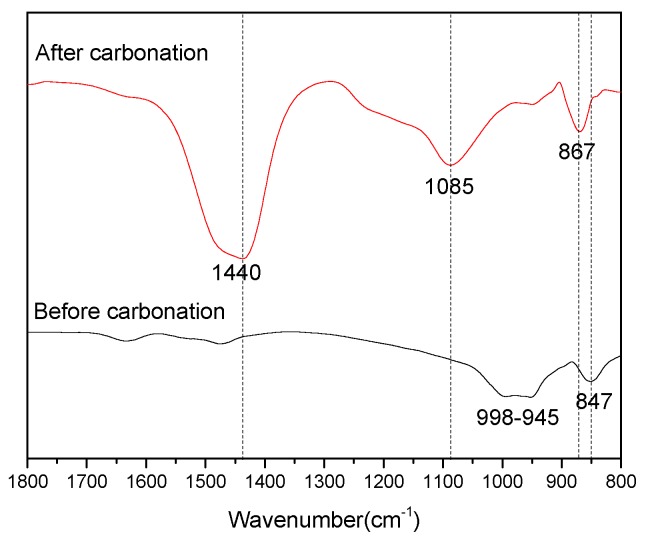
Fourier Transform-Infrared (FT-IR) spectrums of the C_3_S_2_ phase before carbonation and carbonated for 24 h.

To reveal the structure of the SiO_2_ gels formed during carbonation, the FT-IR spectrums of the C_3_S_2_ phase before carbonation and carbonated for 24 h are shown in Figure 3. It is well established that the FT-IR spectrum for silicate compounds exhibit a large absorption between 800 and 1200 cm^−1^, which correspond to the asymmetrical stretching vibration (V_3_) of the Si-O bond. With the increasing polymerization degree of the silicate compound, the bonding strength of the Si-O increases and the V_3_ band shifts to a higher wavenumber. For the C_3_S_2_ phase before carbonation, there were three major absorptions bands located at approximately 847, 945 and 998 cm^−1^. These are higher than the pure C_2_S phase (orthosilicate group), indicating that the C_3_S_2_ phase is composed of dimer silicate tetrahedrons (sorosilicates group), that is, one oxygen atom is shared between two neighboring tetrahedrals. For the C_3_S_2_ phase after carbonation, new bands were observed to appear at approximately 867, 1440 and 1085 cm^−1^. The band located at around 1440 cm^−1^ is due to the asymmetric stretching (V_3_) of the C-O bond present in CaCO_3_, and the band located at around 867 cm^−1^ corresponds to the out of plane bending vibration (V_2_) of the same C-O bond. Moreover, the position of the V_3_ vibration of Si-O bonds were much higher (1085 cm^−1^) than the V_3_ band position present in C_3_S_2_ phase before carbonation, indicating that highly polymerized SiO_2_ gels were formed after carbonation.

#### 3.2.3. TG/DTA Analysis

**Figure 4:**
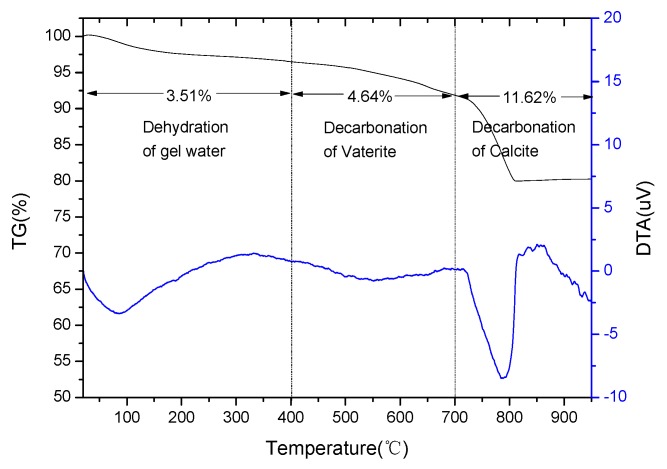
Thermogravimetry and Differential Thermal Analysis (TG/DTA) curves of the C_3_S_2_ phase carbonated for 24 h.

The TG/DTA curves for the C_3_S_2_ phase carbonated for 24 h are presented in Figure 4. The mass losses in the range of 20–400 °C were attributed to the dehydration of the gel water from the formed SiO_2_ gels. The mass losses in the range of 400–700 °C and 700–950 °C were used, respectively, to calculate the mass fraction of vaterite and calcite present in the carbonated C_3_S_2_ phase [19]. The mass losses from the decomposition of vaterite and calcite were 4.64 and 11.62%, respectively, indicating that the CaCO_3_ of C_3_S_2_ phase after carbonation was primarily formed from calcite and some vaterite. These results are consistent with the FT-IR and XRD results.

### 3.3. Microstructure

**Figure 5:**
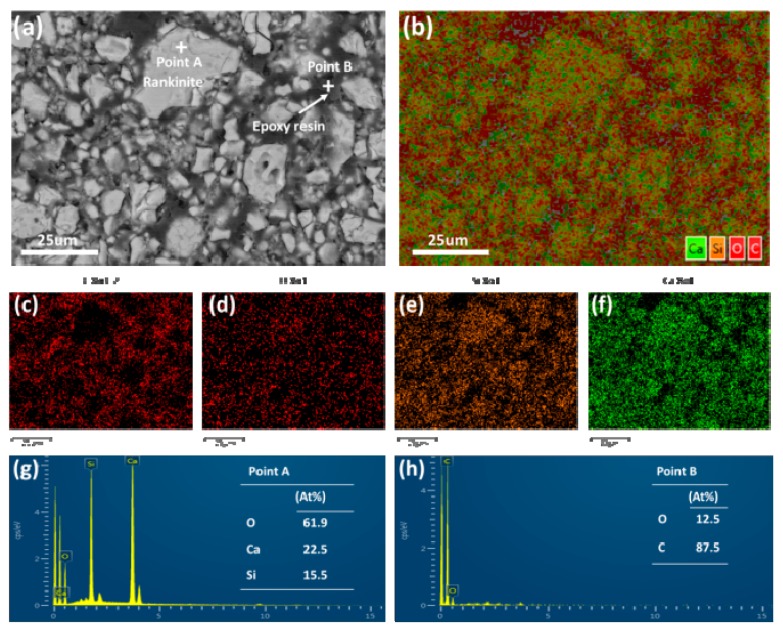
SEM and EDS images of the compacted C_3_S_2_ samples before carbonation: (**a**) Backscattered Electron (BSE) image, (**b**–**f**) elemental maps for composite elements and C, O, Si, Ca, respectively, (**g**,**h**) EDS analysis of point A and B.

**Figure 6:**
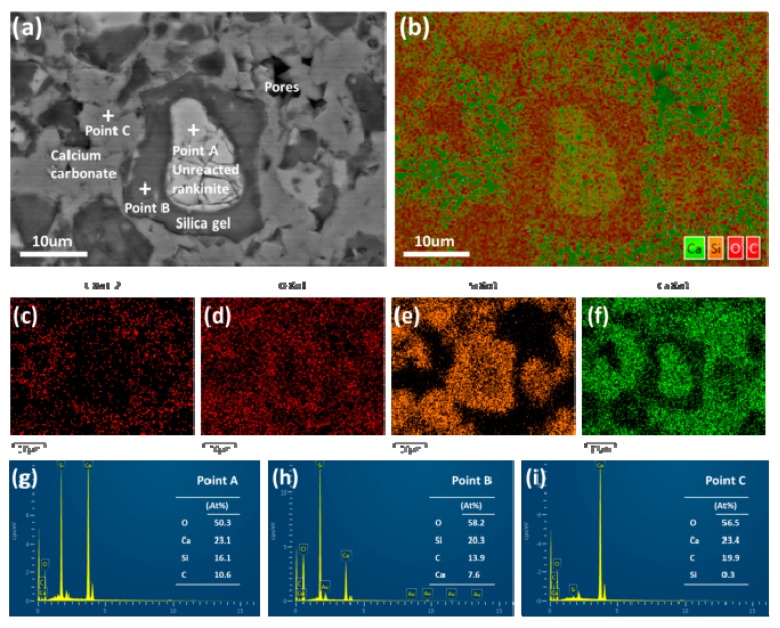
SEM and EDS images of the compacted C_3_S_2_ samples carbonated for 24 h: (**a**) BSE image, (**b**–**f**) elemental maps for composite elements and C, O, Si, Ca, respectively, (**g**–**i**) EDS analysis of point A, B and C.

Figure 5 and Figure 6 show the SEM-EDS images of the compacted C_3_S_2_ samples before and after carbonation, respectively. Before carbonation, the C_3_S_2_ particles (approximately 5–25 μm) were loosely packed. After carbonation, a dense microstructure was observed. According to the elemental maps and EDS results, the distribution of the carbonation products was illustrated as follows. The unreacted C_3_S_2_ core was enveloped by a SiO_2_ gel rim and the initial pores of the sample were filled with CaCO_3_.

### 3.4. Reaction Mechanism

**Figure 7:**
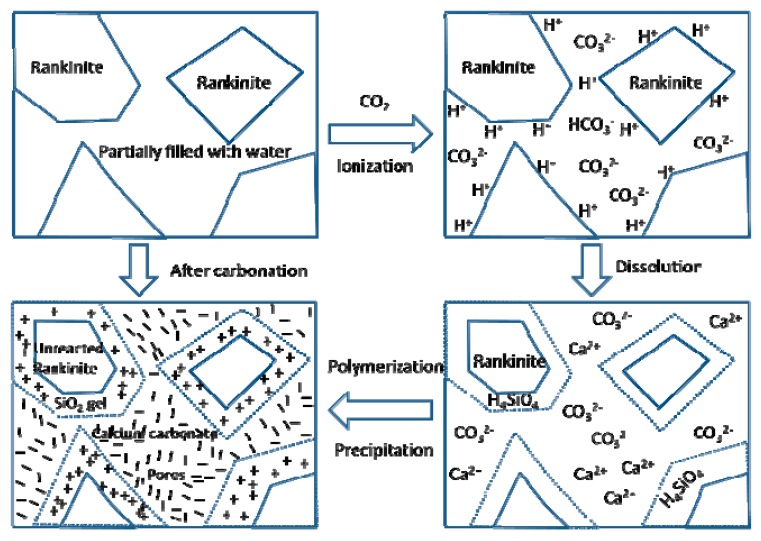
The reaction mechanism that occurred during the carbonation of C_3_S_2_.

Based on the results achieved above, the reaction mechanism that occurred during the carbonation of C_3_S_2_ can be illustrated in Figure 7. When the compacted C_3_S_2_ samples that were partially filled with water come in contact with CO_2_, the CO_2_ will dissolve in the pore water and ionize to produce H^+^, HCO_3_^−^ and CO_3_^2−^.

CO_2_ + H_2_O → H_2_CO_3_ (1)

H_2_CO_3_ → H^+^ + HCO_3_^−^ (2)

HCO_3_^−^ → H^+^ + CO_3_^2−^ (3)

The ionization process of H_2_CO_3_ will generate a lot of H^+^, making the pH value of the pore water fall by approximately 3 units at 20 °C, typically from 7 to 4. Compared with neutral water, the significantly increased H^+^ concentration will induce the solvation of Ca^2+^ from the C_3_S_2_ phase and drive the polymerization of the resulting silicon tetrahedral monomers (H_4_SiO_4_) to form highly polymerized SiO_2_ gels.

6H^+^ + 3CaO·2SiO_2_ + H_2_O → 3Ca^2+^ + 2H_4_SiO_4_ (4)

H_4_SiO_4_ → SiO_2_ (gel) + 2H_2_O (5)

With the progress of dissolution and polymerization, the H^+^ is gradually consumed and the pH of the pore solution is recovered, making it possible to precipitate calcium carbonate. At the beginning, vaterite and aragonite can be formed, but these CaCO_3_ polymorphs eventually revert to calcite. However, in some special circumstances, such as a suitable pH value or specific impurity ions for example, the metastable CaCO_3_ morphology can be stabilized [16]. However, the mechanism by which different polymorphs of CaCO_3_ form during the carbonation process is unclear.

Ca^2+^ + CO_3_^2−^ → CaCO_3_ (6)

In general, the carbonation reaction of C_3_S_2_ can be simplified by combining the above equations. It is important to note that there is no H_2_O in Equation (7). If the silicon tetrahedral monomers are completely polymerized, it is believed that the water plays only a catalytic role in the carbonation reaction process and is not consumed. These results are distinct from the results obtained by Ashraf [18] who believes that water will participate in the carbonation reaction to form C-S-H gels. A possible explanation is that the sample is not completely carbonated. If the C-S-H gels are carbonated completely, the chemically bound water in C-S-H will be released.

3CaO·2SiO_2_ + 3CO_2_ → 3CaCO_3_ + 2SiO_2_ (gel) (7)

As the carbonation reaction proceeds, the pores of the samples are gradually filled with crystalline CaCO_3_ and highly polymerized SiO_2_ gels and the reaction rate is greatly reduced, leaving the unreacted C_3_S_2_ cores. Moreover, it is believed that Ca^2+^ is more mobile than silicon tetrahedral monomers during the carbonation process. Therefore, the highly polymerized SiO_2_ gels remain around the unreacted C_3_S_2_ cores and the CaCO_3_ precipitates in the initial pores. Eventually, a dense microstructure will be formed, which contributes to the excellent mechanical strength.

## 4. Conclusions

Based on the aforementioned results and discussion, the primary conclusions drawn from this work are:

(1) The pure rankinite phase can be easily prepared by calcining the C-S-H gel precursor at a lower temperature. 

(2) The cubic compressive strength of the resulting rankinite reaches a value of 62.5 MPa after 24 h of carbonation curing. 

(3) The main carbonation products formed during the carbonation process are crystalline calcite, vaterite and highly polymerized amorphous silica gels. 

(4) The formed carbonation products fill the pores and bind to each other, creating a dense microstructure which contributes to the excellent mechanical strength. 

(5) The results provide a novel insight into potential recycling of waste concrete powders for prefabricated building products with lower CO_2_ emissions.
